# The biology of extracellular vesicles with focus on platelet microparticles and their role in cancer development and progression

**DOI:** 10.1007/s13277-016-5358-6

**Published:** 2016-09-15

**Authors:** M. Żmigrodzka, M. Guzera, A. Miśkiewicz, D. Jagielski, A. Winnicka

**Affiliations:** 1 Department of Pathology and Veterinary Diagnostics, Faculty of Veterinary Medicine, Warsaw University of Life Sciences (WULS-SGGW), Nowoursynowska 159c, Warsaw, Poland; 2 Department of Veterinary Medicine, University of Cambridge, Madingley Road, Cambridge, UK; 3 Veterinary Clinic BIALOBRZESKA, Częstochowska 20, Warsaw, Poland

**Keywords:** Extracellular vesicles, Microvesicles, Exosomes, Platelet microparticles, Cancer

## Abstract

Extracellular vesicles (EVs) are a heterogeneous group of structures which can be classified into smaller in size and relatively homogenous exosomes (EXSMs)—spherical fragments of lipid bilayers from inner cell compartments—and bigger in size ectosomes (ECSMs)—a direct consequence of cell-membrane blebbing. EVs can be found in body fluids of healthy individuals. Their number increases in cancer and other pathological conditions. EVs can originate from various cell types, including leukocytes, erythrocytes, thrombocytes, and neoplastic cells. Platelet microparticles (PMPs) are the most abundant population of EVs in blood. It is well documented that PMPs, being a crucial element of EVs signaling, are involved in tumor growth, metastasis, and angiogenesis and may participate in the development of multidrug resistance by tumor cells. The aim of this review is to present the role of PMPs in carcinogenesis. The biology and functions of PMPs with a particular emphasis on the most recent scientific reports on EV properties are also characterized.

## Introduction

Extracellular vesicles (EVs) are released under multiple physiological conditions, e.g., cell maturation and aging, by a variety of cells. Their presence was detected in a number of body fluids of healthy individuals, including peripheral blood, urine, saliva, semen, cerebrospinal fluid, synovial fluid, bronchoalveolar lavage, and bile [1, 2]. The mechanism of EV formation and their biochemical composition depends on the type and function of cells from which they originate [3]. EVs express antigens typical of cells from which they are released and they serve as carriers of many bioactive molecules, including proteins, mRNA, and miRNA. Therefore, they play an important role in cell-to-cell communication.

In 1967, Peter Wolf first identified small procoagulant structures deriving from activated platelets in human blood and initially termed them “platelet dust” [3, 4]. This finding was a milestone in EV research allowing further studies on their role and function in various disease conditions.

Platelet microparticles (PMPs) are the most abundant microparticle population in peripheral blood, accounting for around 70–90 % of all EVs [5–7]. An increase in the number of PMPs was observed in neurological diseases [2, 8], thrombotic disorders, primary immune thrombocytopenia, uremia, and lymphoma [5]. It has been shown that PMPs are actively involved in angiogenesis [5, 9, 10]. Currently, much attention is paid to the potential role of EVs, including PMPs, in cancer progression [11]. It has been suggested that PMPs are able to promote growth of a primary tumor, can stimulate angiogenesis, and contribute to the formation of distant metastases. Nevertheless, the basic mechanisms of these phenomena still need to be elucidated.

## Terminology and classification

EVs are a heterogeneous group of predominantly spherical structures being released by eukaryotic and prokaryotic cells, both, in vivo and in vitro [12]. Their production increases during cell activation, oxidative stress, tissue hypoxia, and in various disease conditions [11, 13, 14]. Lack of standardization of definitions deriving from the broad literature on EVs results in inconsistency in the EV classification schemes. The absence of unification is likely a consequence of variability in EVs’ size (from 30 nm to 1 μm) and their diverse cellular origin. EVs can be divided according to their size into ectosomes (ECSMs), also called microparticles (MPs) or microvesicles (MVs), that vary in size between 0.1 and 1 μM, and exosomes (EXSMs) in size of 30–100 nm. EVs are more commonly classified using their cellular origin. Using the latter criterion, the following EV categories are distinguished: platelet microparticles (PMP), erythrocyte microparticles (RBC-MPs), endothelial cell microparticles (EMPs), and tumor cell microparticles (TMPs) [15]. Oncosomes are another type of EVs. These particles are much bigger (1–10 μm) than the ECSMs, are capable of active movement, and originate from migrating tumor cells [16–18].

EXSMs are relatively homogenous spherical fragments of lipid bilayers of the inner cell compartments [19, 20], containing proteins, mRNA, microRNA, and lipids [12, 21]. Exosomes deriving from reticulocytes were first described as released extracellularly intraendosomal vesicles by Johnstone in 1987 [21, 22]. Further studies performed on EXSMs in the 80s demonstrated their role in physiological processes, including participation in erythrocyte maturation by eliminating some of erythrocyte surface membrane receptors [1, 22]. Exosomes can derive from various cell types (e.g., platelets, lymphocytes, astrocytes, fibroblasts, and neoplastic cells) [1, 23, 24] and can be classified according to their functions [25]. Some exosomes participate in antigen presentation and stimulate immune response, while the ones containing RNA are involved in intercellular communication [26, 27].

ECSMs, other than exosomes, are more diverse and they form during cell activation due to plasma membrane blebbing [12, 16, 21, 28]. All MPs are formed directly from the cell membrane, similar to apoptotic bodies [29, 30] that also belong to EVs. However, they can be distinguished from ectosomes and exosomes due to their different biogenesis and structures. Apoptotic bodies are 1–5 μm in diameter and they form by cell-membrane blebbing when the cell undergoes apoptosis; thus, they may contain the nuclear fragments [15, 31–33].

Some authors postulate that the term “platelet-derived microparticles” should be reserved only for particles between 0.05 and 1 μm in size [33]. Larger may be confused with platelets or PMPs’ aggregates [34], while the smaller ones (between 0.04 and 0.08 μm), may be misclassified as exosomes originating from platelet granules [33, 34]. PMPs comprise up to 70–90 % of all MVs present in the circulation. About 10 % of them are derived from granulocytes and only 5 % from monocytes, red blood cells, or endothelial cells [4–6, 35]. Previous reports have also shown that, both, megakaryocytes and immature platelets are able to form MVs [35, 36].

## Mechanisms of EV biogenesis

**Fig. 1:**
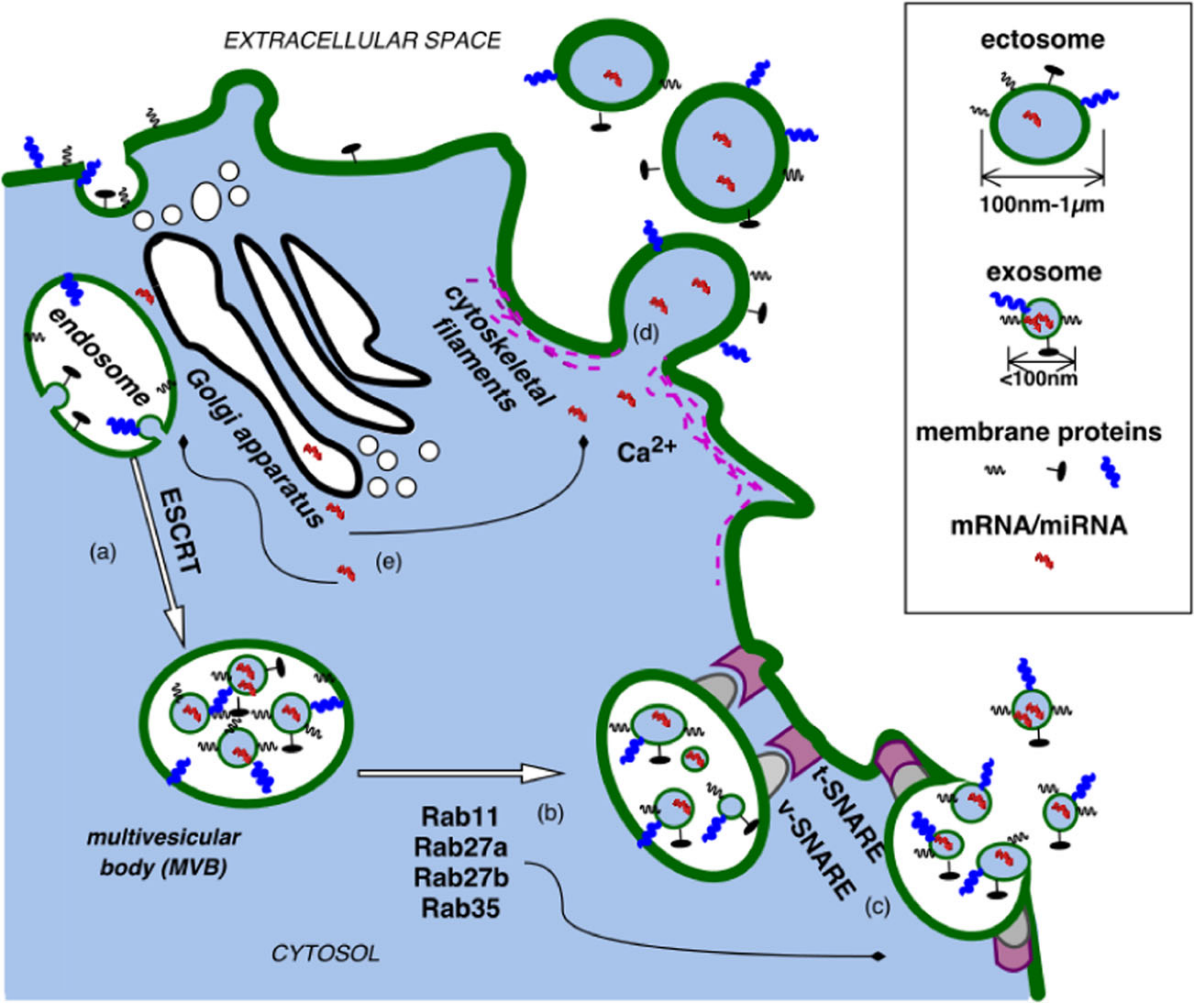
Formation of exosomes (EXSMs) and ectosomes (ECSMs)

The mechanism of EV formation and their transport to the extracellular space vary depending on their type [21, 37, 38] (Fig. 1.). The mechanism of MV formation from endothelial cells was described for the first time by Biscoe and Stehbens in 1966. Their discoveries were followed by observations made by Wolf [39]. The postulated mechanism was based on fenestration and permeability of endothelial cells of the arterioles in the carotid glomus. Over three decades later, in vitro studies have proven that EVs can be also formed from venous endothelial cells [40]. The exact, likely cell-type specific, mechanism of their formation through cell membrane blebbing has not yet been entirely clarified indicating the need of further studies. On a more general level, EV formation involves vertical translocation of phospholipids—phosphatidylserine (PS) and phosphatidyl-ethanolamine (PE)—and engages actin filaments (AFs) [7, 41].

### EXSM formation

During the formation of EXSMs, cell membrane bulges into the lumen of a late endosome creating a structure called multivesicular body (MVB) [21, 37]. Endosomal sorting complex required for transport (ESCRT) participates in sorting of MVB’s content and EXSMs in the cytosol [21], while the members of Rab family proteins (i.e., Rab11, Rab27a, Rab27b, Rab35) are involved in the transport, fusion, and secretion of EXSMs [21]. Also the transmembrane protein complex SNARE (soluble N – ethylmaleiamide sensitive factor attachment protein receptor) has been shown to be responsible for the release of EXSMs into the extracellular space [21, 23, 42]. In fact, the structure and chemical composition of EXSMs are not the same as of the parental cell membrane. When EXSMs form, PS is transferred to the outer lipid layer and exposed at the site of membrane fusion. In the same time, the distribution of membrane proteins remains unchanged [21]. EXSMs contain proteins and/or nucleic acids (mRNA, microRNA) specific for their progenitor cells but never all proteins of the parental cell membrane are present on exosomes [30, 43].

### ECSM and PMP formation

ECSMs are formed by cell-membrane budding. Physiologically phospholipids of the cell membrane are arranged asymmetrically: phosphatidylcholine (PC) and sphingomyelin (SM) are present in its outer layer, while PS and PE in the inner layer. This arrangement is controlled by a group of enzymes, such as flippase, aminophospholipid translocase, floppase, and scramblase [21]. Flippase is responsible for the transfer of PE and PS from the outer to the inner layer of the cell membrane. Floppase has been shown to have an opposite effect. Their activity is regulated by ABCC1 protein, also known as a multidrug-resistance protein 1 (MRP1) [44]. By contrast, lipid transport is determined by scramblase. Its deficiency is a rare congenital bleeding disorder associated with platelet dysfunction named Scott’s syndrome [45]. The translocation of PS and PE within the lipid bilayer requires energy from ATP hydrolysis [43].

Calcium ions play an essential role in the process of MP formation. Increased intracellular calcium which is secondary to its release from endoplasmic reticulum, inactivates flippase and activates floppase. This leads to reorganization of phospholipids in the cell membrane due to degradation by Ca^2+^-dependent proteolysis [2, 43]. An activation of scramblase requires greater increase in the calcium concentration and is therefore considered as less important for the formation of MPs [46]. During ECSM formation the activation of calpain and gelsolin occurs. The ability of the latter one to bind and partially degrade actin filaments leads to breaking of bonds between the cytoskeleton filaments and phospholipids. The weakening of the protein fibrils of the cytoskeleton initiates ECSM budding [43, 47].

Platelet activators, such as thrombin, collagen ADP, and Ca^2+^ ionophore, activate resting platelets to shed PMPs [3, 4, 48]. The effect of calcium ions and calpain on the formation of PMPs was demonstrated using platelet activators and their inhibitors, including thrombin, collagen, and dibucaine [3, 49–51]. Weidmer et al. have shown that preincorporation of a calpain inhibitor called leupeptin into platelets totally blocked C5b-9-induced proteolysis of talin, myosin, and actin binding protein (ABP), having no effect on platelet secretory activity and formation of PMPs. These data show that formation of PMPs in response to C5b-9 is dependent upon an inflow of calcium into the platelet cytosol which does not require metabolic energy or calpain-mediated proteolysis of cytoskeletal proteins [52]. Protein tyrosine dephosphorylation [53] and calmodulin activation [54] are also involved in the formation of PMPs; however, their role is less clear [3].

PMPs found in peripheral blood express CD41 [5] and have a short half-life [3, 5]. They are constantly shed into the circulation in certain amounts even in healthy patients [3, 55]. It is believed that some fraction of circulating PMPs, named megakaryocyte-derived microparticles (MKMPs), originates from megakaryocytes [5]. In vitro megakaryocytes form MKMPs at a diameter of 0.1–0.3 μm [3]. PMPs are also released during the storage of platelet concentrates. This process can be inhibited by administration of platelet activation inhibitors which does not affect the platelet count [3, 56].

## EV structure and components

A hallmark of all EVs is the expression of surface proteins specific for or associated with their parental cells [57]. The basic set of proteins located on EXSMs consists of tetraspanin family proteins (CD9, CD63, CD81, and CD82), lipid-binding surface protein—milk fat globule EGF/factor VIII (MFGE8), MHC class I and II molecules, proapoptotic-apoptosis-linked genes 2 (ALG-2), thioredoxin peroxidase 2 (TPxII) and anti-apoptotic (galantine 3) proteins [58, 59], surface peptidases (CD13, CD26), and protein from tumor susceptibility gene 101 (TSG101) involved in the MVB biogenesis [27, 59]. EXSMs also contain many other proteins, such as annexins, clathrins, heat shock proteins (HSP), and cytoskeletal proteins (actin, myosin). Enzymes present in EXSMs include enolases, phosphoglycerate kinase, aldolases, and glyceraldehyde 3-phosphate dehydrogenase (GAPDH). Moreover, they contain translation initiation factor—eIF4E—and elongation factor—eEF1 [28]. EXSMs’ protein composition and function prove that rather than being only spherical fragments of the cell membrane they should be considered as subcellular compartments formed in a specific and planned fashion [27].

MPs are less homogeneous structures. Like EXSMs, they contain numerous markers that determine their origin, e.g., CD41 for platelets, CD235a for erythrocytes, and CD11c for dendritic cells [43, 60]. Their membranes are built out of phospholipids which have similar composition to those of the parental cells, particularly their lipid rafts. However, their membrane profile has not been determined as accurately as the one of EXSMs [30]. On the other hand, MVs secreted from B cells, dendritic cells, and melanoma cell lines are richer in sphingomyelin, rather than in cholesterol which is characteristic for their parental cells [61].

MPs as EXSMs also contain RNA which can mediate genetic communication between cells, for instance, pancreatic cancer cell lines release PMPs containing mRNAs and microRNAs [21, 62]. Valadi and colleagues have shown that exosomes derived from human and mice mast cell lines contain both mRNA and miRNA. The mRNA carried by exosomes was functional, which means it is capable of encoding polypeptides in support of protein synthesis. Interestingly, some miRNAs that they have found in exosomes were expressed at higher levels than in the donor cells. Moreover, they have demonstrated that following the incubation of human cells together with mouse exosomes, some mouse proteins were found in the recipient cells. The presence of RNA in exosomes suggests that they act like vehicles for the gene-based communication between cells; thus, they may modulate recipient-cell protein production [62].

### PMP structure and components

PMPs contain more than 40 glycoproteins characteristic of platelets [4, 63–65], including surface markers GP IIa/IIIa (CD41) and GP Ia/IIa (CD49b/CD29), P-selectin, (CD62), gp53 (CD63), and receptor present on activated platelets (receptor of activated glycoprotein IIb/IIIa PAC-1) [4, 66–70]. During activation, platelets release many bioactive substances, normally stored in their dense and α granules. Holme et al. have shown that PMPs formed after platelet activation by calcium ionophore A23187 contain many proteins characteristic for α granules of activated platelets, such as thrombospondin, platelet factor 4, and β-thromboglobulin [4, 71]. Platelet activating factor (PAF), normally released from activated platelets under the influence of collagen or thrombin, was found to be related to PMPs in 90 % [4, 72]. In mice with hemophilia A, PMPs increased after P-selectin infusion [73], whereas in patients treated with recombinant activated factor VII (rFVIIa) a temporary increase in the release of PMPs occurred [74]. PMPs are also able to bind fibrinogen and participate in thrombus formation [75].

PMPs’ density depends largely on the qualitative and quantitative composition of glycoproteins [76, 77]. The majority of glycoproteins located in the cell membrane consist of adhesion receptors, membrane transporters, and metalloproteinases (MMPs). MMPs represent a large group of proteolytic enzymes that are able to degrade the components of extracellular matrix, thus, promoting cancer progression, facilitating tumor growth, angiogenesis, and metastasis [76, 78–80]. The increased activity of MMPs has been demonstrated in many tumor types, for instance, membrane-type matrix metalloproteinases (MT-MMPs) were shown to be overexpressed in lung cancer [79, 81–83].

PMPs’ function is multidirectional and their effect depends on target cell type. [15]. They induce monocyte chemotaxis, cause an increase in tissue factor (TF) expression on the surface of endothelial cells, and influence adhesion and proliferation of normal and neoplastic hematopoietic cells. They have been also shown to be involved in HIV infection [69, 70, 84–87].

## PMP elimination

Platelets circulate in human blood for about 10 days. PMPs injected into mice peripheral blood are cleared from circulation within 20–60 min (unpublished observation of Flaumenhaft) [3, 15]. Their elimination from the circulation occurs in several manners. PMPs can be removed by phagocytosis after their previous opsonization with complement proteins, Gas6, thrombospondin, and S protein. Flaumenhaft has shown that microparticles are opsonized by C3b complement component [3, 63, 82, 88]. Alternatively, when opsonization is facilitated by PS externalization, the process can also occur with the involvement of the C3 component [3, 89–91]. When PMPs are released, they remain in the circulation for only a certain period of time because they fuse with target cells and because they may be phagocytized by macrophages, as PMPs express PS on their surface. It has been shown that following PS binding with annexin V, the fusion of PMPs with target cell is blocked that prevents exchange of their cargo [15]. PMPs may also be eliminated by serum phospholipase A2 activity [3, 92]. It is still unclear which of the abovementioned mechanisms is the most significant mean of PMP elimination. Recently, new PS receptors have been described, including Tim1 (T cell immunoglobulin mucin 1), stablin 2, and BAI1 (brain-specific angiogenesis inhibitor 1), and some of them may be involved in the cellular uptake of MVs with PS expression [15].

## Biological functions of EVs

Many authors emphasize the role of EVs as transcellular signal delivery particles [66, 67, 93, 94]. It can be hypothesized that this way of cellular communication is a consequence of eukaryote evolution and could have preceded the soluble mediator-based mechanism [69]. Small size of EVs facilitates long distance movement within body fluids.

The composition of EVs reflecting their cellular origin allows them to transmit specific signals. They can bind to various target cells exhibiting broad spectrum activity [69]. It is thought that due to the diversity and the amount of transmitted information, this type of intercellular communication can play a crucial role in the modulation of the surrounding microenvironment [30, 42]. EVs are able to stimulate target cells directly by providing ligands which increase the secretion of various signaling molecules, e.g., growth factors or cytokines [70, 95, 96]. They can also transfer membrane receptors and adhesion molecules [97]. Furthermore, EVs deliver proteins, mRNA, and transcription factors causing epigenetic reprogramming of target cell [98]. A cell which has internalized EVs can undergo functional transformation and can start to communicate with the microenvironment in the way it was programmed by the engulfed particle [11].

## Effects of EVs on the immune system

EXSMs first attracted interest of immunologists in 1996 when it was found that B lymphocytes transformed by Epstein-Barr virus were able to secrete EXSMs by the fusion of MVBs with the plasma membrane. Secreted EXSMs were shown to play a role in antigen presentation [1, 21]. MVs may act as vectors for many pathogens and prions [99–101]. MV-associated HIV transfer is enabled via chemokine receptor CCR5 which is used by macrophage-tropic HIV-1 to access CCR5+ peripheral blood mononuclear cells. This receptor is also expressed on MVs secreted by peripheral blood mononuclear cells and can be transferred by MVs to CCR5− cells. This transfer allows the virus to penetrate target cells [102]. Similar observation has been made by Rozmyslowicz and colleagues. They have demonstrated that peripheral blood PMPs and megakaryocyte-derived MPs can transfer the CXCR4 chemokine receptor from the surface of platelets or megakaryocytes to the surface of CXCR4-null cells that render them more susceptible for T tropic virus infection (X4 HIV strains, for which CXCR4 is essential to enter the cell) [94]. In hepatitis C virus infection, MVs facilitate the transfer of CD81 which is the crucial co-receptor for B and T cell activation [103].

EXSMs secreted by antigen-presenting cells (APCs) can be recognized by CD4+ and CD8+ T cells [1]. Studies on EXSMs deriving from dendritic cells (DCs) showed their ability to stimulate CD8+ T cells in MHC class I-dependent manner. The stimulation with EXSMs was greater when they derived from mature DCs in comparison with the ones originating from the more immature cells [104]. EXSMs secreted by B lymphocytes induce MHC class II-dependent T cell response [1].

EXSMs secreted by tumor cells, the so-called tumor extracellular vesicles (TEVs), were found to modulate the immune response by inhibition of DC differentiation, NK cytotoxic activity, as well as by promoting the expansion of immunosuppressive T regulatory lymphocytes, activated macrophages, or NK cells [1, 21]. Their immune-modulatory abilities may find application as a novel therapeutic approach especially in neoplastic diseases.

### PMPs and the immune system

PMPs also have pro-inflammatory effects and may contribute to the development of certain diseases, such as atherosclerosis [9]. PMP increase has been observed in patients with acute coronary syndromes during coronary angioplasty, diabetes [86, 105], and Alzheimer’s disease [106]. Sprague et al. showed that PMPs transfer CD154 (CD40L) leading to B-cell activation [5, 107]. Other studies confirmed modulatory properties of PMPs at the site of inflammation, including their ability to induce chemotaxis of monocytes, NK cells, and lymphocytes [108]. Due to CD41 and CD62P expression, they may activate neutrophils and endothelial cells emphasizing their important role in inflammatory processes [109]. PMPs secreted from activated platelets transfer CD41 membrane receptor enhancing the adhesion of hematopoietic cells to fibrinogen [5, 66]. Furthermore, due to the expression of sphingosine 1-phosphate (S1P), they can induce proliferation of endothelial cells [11, 110]. Also the presence of arachidonic acid (AA) on their surface allows them to show pro-angiogenic properties [11, 111–113].

## PMPs in cancer development and progression

**Fig. 2:**
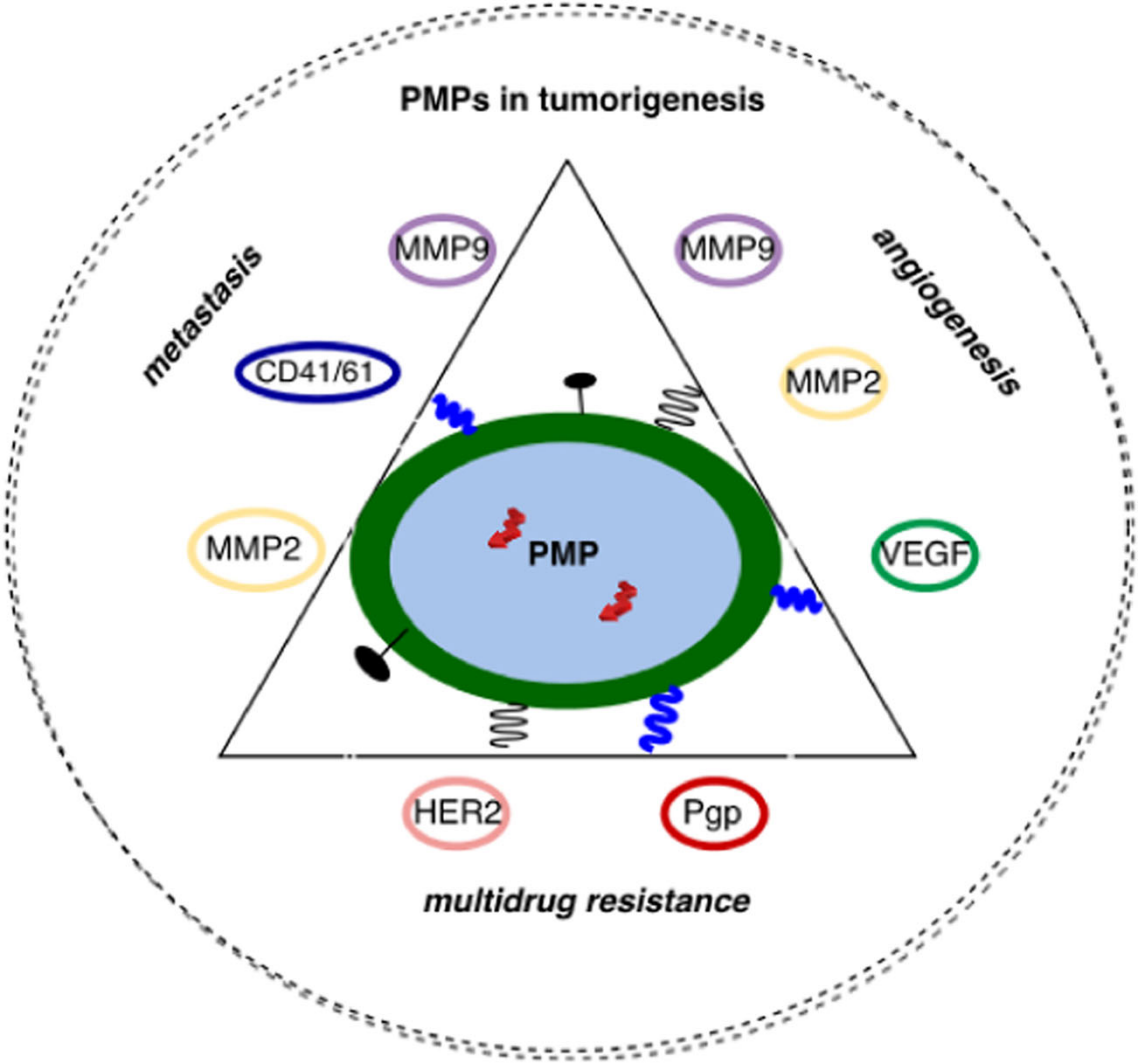
Platelet microparticles (PMPs) in tumorogenesis

The role of EVs in cancerogenesis has been demonstrated in numerous studies and the most attention was brought to EXSMs, PMPs, and TMVs. Presumably, the abovementioned particles are involved in tumor growth, metastasis, and angiogenesis (Fig. 2). Janowska-Wieczorek et al. evaluated the role of PMPs in the tumor development and metastasis using five cell lines of human lung cancer and murine Lewis lung carcinoma cell line (LLC) [67]. Authors have used flow cytometry to demonstrate that the expression of glycoprotein GPIIb/IIIa (CD41), specific for platelets and expressed on PMP surface, was also found on human lung cancer cell lines. It shows that PMPs can transfer the receptor to the surface of cancer cell in vitro [67]*.* Moreover, PMPs were able to stimulate kinase-dependent protein phosphorylation (MAPK p42 / 44 and AKT) and increase the expression of matrix metalloproteinases—Type 1 (MT1-MMP) [67]. Authors assessed the ability of PMP internalization by tumor cells, their impact on cell activation, and the type of signal pathways involved in this process [67]. The effect on cell proliferation, expression of angiogenic factors (including MMPs), and chemoinvasion was also evaluated [67]. PMPs induced chemotaxis in four of the cell lines tested. Particles also increased the A549 cell line proliferation resulting in the formation of distant lung metastases and abnormal expression of cyclin D2 in C57BL/6 mice in vivo [67].

PMPs have the ability to induce the expression of mRNA of MMP-9, vascular endothelial growth factor (VEGF), and interleukin 8 (IL-8) [67]. The administration of PMP-coated cells (LLC) to mice resulted in an increase in the distant metastasis to the bone marrow and lungs comparing with the control group receiving only the LLC cells [67, 114]. These observations indicate the participation of platelets and PMPs in tumor progression, metastasis, and angiogenesis [115–117].

A different group of researchers found that PMPs have a consistent anti-apoptotic effect on human umbilical vein endothelial cells (HUVEC) which depended on the concentration of thrombin [118]. It has been also shown that this mechanism is controlled by the processes crucial for apoptosis induction. Initially, annexin I located on the EMPs participates in their internalization into endothelial cells through the receptor for phosphatidylserine [118]. Next, inhibition of apoptosis involves blocking of p38 activation and induces activation of MAPK phosphatase 1 (MKP-1) and internalization of EMPs which carries the anti-apoptotic and pro-angiogenic microRNA (miR-126, miR-296) [119, 120]. Nonetheless, it is still unclear whether internalization of EMPs occurs via fusion or phagocytosis [118].

Formation of distant metastases requires from the tumor cells to get through the event cascade that consists of angiogenesis, passage through the vessel wall, survival in the circulation, and finally proliferation at the site of newly forming metastases. Bakewell et al. proposed that integrin β3 (heterodimer of αIIbβ5 and α_V_β_3_) plays an essential role in the formation of metastases [114]. Their further studies confirmed that mice platelet receptor (GP IIb/IIIa) antagonists play a protective role in the formation of metastases in bones and other organs [114] due to their inhibitory properties on the interaction between the platelets and tumor cells, but also platelets themselves [114]. Tumor cell-platelet interactions leading to metastasis are based on the platelet’s ability to adhere to the damaged vascular endothelium, as well as their ability of paracrine regulation of tumor cell proliferation and growth. Platelets also can protect circulating neoplastic cells from the cells of immune system. Considering the above, PMPs’ contribution to metastasis via similar manner, is likely.

EVs may also participate in the development of multidrug resistance by tumor cells by transferring P-glycoprotein (P-gp) [121, 122] or by inducing the expression of human epidermal growth factor receptor 2 (HER2) on their surface. That may reduce the efficacy of anti-HER2 therapeutic antibody (Trastuzumab) in breast cancer [121, 123]. MVs can transfer angiogenic factors intracellularly or pericellulary. They may induce the expression of pro-angiogenic genes through a direct cellular contact, e.g., with endothelial cells [15, 70, 124, 125]. PMPs’ properties, such as presence of pro-angiogenic factors from α granules like VEGF, platelet-derived growth factor, fibroblast growth factor, and metalloproteases, are determined by their platelet origin [15, 35, 70]. Kim et al. evaluated PMPs’ contribution to angiogenesis focusing on their impact on endothelial cell proliferation and survival in vitro [35]. Other in vivo and ex vivo studies have shown that PMPs stimulate progenitor cells to form a capillary network [35, 126, 127]. Moreover, MVs secreted by T cells and endothelial cells modulate angiogenesis, while PMPs stimulate the secretion of pro-angiogenic factors by tumor cells [35, 128]. The role of TF in tumor growth, angiogenesis, and metastasis is well documented; therefore, it is not surprising that its presence on PMPs’ surface was found to facilitate metastasis [35, 129].

## Conclusions and future directions

Few preliminary reports suggest only certain benefits of EV-based therapy. TMPs with high expression of MDR can transfer nucleic acids to MDR (−) tumor cells and can cause drug resistance. Thus, pharmacologically reduced release of TMPs represents one of the strategies of anti-cancer therapy. Another idea is to harness the anti-cancer drug-loaded MVs. It has recently been shown, in a syngeneic mouse model of CT26 colorectal cancer, that intravenous injection of EVs loaded with doxorubicin is an effective way to inhibit tumor growth [130]. Further studies that may reveal other benefits of this therapeutic approach are warranted.

Numerous studies have been conducted to assess the possibility of PMP application in the treatment of various diseases. In patients with hormone-refractory prostate cancer (HRPC), high level of PMPs was correlated with aggressiveness of the tumor growth and poor prognosis. While in non-small cell lung cancer patients, their high levels before and after the treatment correlated well with prolonged survival [5, 16, 131]. Moreover, in patients with gastric cancer, PMP level is a better prognostic indicator than IL-6 or VEGF [5, 132]. Although still not used in routine diagnostic PMPs are regarded as significant prognostic factors in oncological patients.

Despite the small size of EVs, the ability of their formation by different cell types, the ease of their travel through the body, and the magnitude of information they carry make them a significant factor which regulates the immune response and modulates the tumor microenvironment. It is well documented that EVs and PMPs participate in signal transduction into the cells, including the neoplastic one, thereby modulating their function. In view of their components, functions, and bioavailability, EVs have been considered as a potentially great vehicle for the administration of drugs and different molecules, including RNA, to modulate cell activity in many disorders such as cancer. However, to make EVs applicable and efficacious in the treatment, some of their underlying functions still need to be better understood.
